# Characterization of key triacylglycerol biosynthesis processes in rhodococci

**DOI:** 10.1038/srep24985

**Published:** 2016-04-29

**Authors:** Sawsan Amara, Nicolas Seghezzi, Hiroshi Otani, Carlos Diaz-Salazar, Jie Liu, Lindsay D. Eltis

**Affiliations:** 1 Department of Microbiology and Immunology, Life Sciences Institute, The University of British Columbia, 2350 Health Sciences Mall, Vancouver, BC V6T 1Z3, Canada

## Abstract

Oleaginous microorganisms have considerable potential for biofuel and commodity chemical production. Under nitrogen-limitation, *Rhodococcus jostii* RHA1 grown on benzoate, an analog of lignin depolymerization products, accumulated triacylglycerols (TAGs) to 55% of its dry weight during transition to stationary phase, with the predominant fatty acids being C16:0 and C17:0. Transcriptomic analyses of RHA1 grown under conditions of N-limitation and N-excess revealed 1,826 dysregulated genes. Genes whose transcripts were more abundant under N-limitation included those involved in ammonium assimilation, benzoate catabolism, fatty acid biosynthesis and the methylmalonyl-CoA pathway. Of the 16 *atf* genes potentially encoding diacylglycerol *O*-acyltransferases, *atf8* transcripts were the most abundant during N-limitation (~50-fold more abundant than during N-excess). Consistent with Atf8 being a physiological determinant of TAG accumulation, a Δ*atf8* mutant accumulated 70% less TAG than wild-type RHA1 while *atf8* overexpression increased TAG accumulation 20%. Genes encoding type-2 phosphatidic acid phosphatases were not significantly expressed. By contrast, three genes potentially encoding phosphatases of the haloacid dehalogenase superfamily and that cluster with, or are fused with other Kennedy pathway genes were dysregulated. Overall, these findings advance our understanding of TAG metabolism in mycolic acid-containing bacteria and provide a framework to engineer strains for increased TAG production.

Rhodococci are mycolic acid-producing Actinobacteria that are of burgeoning importance in environmental and biotechnological applications due in part to their ability to catabolize a remarkably wide range of organic compounds^1^. Many strains of *Rhodococcus* genus are oleaginous, producing and accumulating large quantities of triacylglycerols (TAGs). TAG accumulation occurs under conditions of carbon excess when the bacterium is subject to non-carbon nutritional stress, such as nitrogen limitation^2^. TAGs can constitute up to 85% of the cellular dry weight (CDW)^3^,^4^ and are stored as lipid droplets (LD), organelle-like structures with defined protein composition^5^. The oleaginous nature of rhodococci has taken on new importance given the potential of these strains to degrade lignocellulosic biomass^6^,^7^,^8^ and the potential of TAGs as a feedstock for biotechnological applications such as feed additives, cosmetics, oleochemicals, lubricants and biofuels^9^,^10^.

In rhodococci and other Actinobacteria, TAG biosynthesis occurs via the Kennedy pathway (Fig. 1)^11^. In this pathway, *sn*-glycerol–3-phosphate is sequentially acylated with fatty acyl-CoAs by glycerol-3-phosphate *O*-acyltransferase (GPAT, encoded by *plsB*) and 1-acylglycerol–3-phosphate *O*-acyltransferase (AGPAT, encoded by *plsC*) to yield phosphatidic acid. Phosphatidic acid is then dephosphorylated to diacylglycerol (DAG), in a reaction proposed to be catalyzed by a type 2 phosphatidic acid phosphatase (PAP2)^12^. In the final step, *sn*-3 acylation of the DAG is catalyzed by a wax ester synthase/DAG *O*-acyltransferase (WS/DGAT) encoded by an *atf* gene. Despite this knowledge, efforts to understand TAG biosynthesis in rhodococci have been complicated by the occurrence of multiple homologs of Kennedy pathway enzymes in these bacteria and the lack of biochemical or molecular genetic characterization.

The multiplicity of TAG biosynthetic enzymes is exemplified by the number of predicted WS/DGATs in *Rhodococcus opacus* PD630 and *Rhodococcus jostii* RHA1: PD630 contains 17 *atf* homologs^13^ and RHA1 contains 16^2^ including three homologs of *atf14* whose gene products share 98% amino acid sequence identity^14^. The homologs are numbered differently in the two strains, but are numbered here according to RHA1 unless otherwise indicated (Fig. 1). Deletion of either *atf1*_PD630_ or *atf2*_PD630_ of PD630, corresponding respectively to *atf3* and *atf6* in RHA1, resulted in a 30–50% decreased fatty acid (FA) content during stationary phase compared to the wild-type using gluconate as a growth substrate^13^,^15^. Nevertheless, Atf1_PD630_ appears to be a WS based on its activity in *Escherichia coli* extracts while Atf2_PD630_ had a higher DGAT activity^15^. Transcriptomic studies in PD630 further indicated that the homologs of Atf6, Atf8 and Atf10 are highly expressed during TAG accumulation^16^. The Atf6 and Atf8 homologs were further implicated in TAG accumulation by virtue of their association with LDs. Interestingly, the homolog of Atf9 was highly repressed during TAG accumulation^16^. These results are consistent with recent proteomic studies of RHA1 which revealed that Atf6, Atf8 and Atf10 are more abundant in RHA1 under lipid storage conditions^17^. Nevertheless, the precise roles of the different WS/DGATs in wax ester (WE) and TAG biosynthesis, respectively, remain largely unknown.

Proteomics and transcriptomics studies have identified a number of other genes involved in TAG biosynthesis and have begun to provide a more integrated view of this biosynthesis with respect to cellular metabolism. Whole cell proteomic studies in gluconate-grown RHA1 have indicated that a number of metabolic pathways are up-regulated during TAG accumulation including the Entner-Doudoroff pathway, the pentose-phosphate shunt, branched-chain amino acid catabolism and the methylmalonyl-CoA pathway^17^. Upwards of 261 genes have been implicated in the metabolism of TAGs in PD630 based on metabolic reconstruction and bioinformatic analyses^14^. Proteomic studies have also identified 228 LD-associated proteins in RHA1, the two most abundant of which were RHA1_RS10270 (formerly Ro02104) and PspA^5^. The former includes a predicted apolipoprotein domain and was annotated as “microorganism lipid droplet small” (MLDS) because its deletion yielded larger LDs. Similarly, 177 LD-associated proteins were found to be differentially produced under lipid-accumulating conditions in PD630, including the MLDS homolog^16^, previously identified as TadA^18^. In these studies, the LD-associated proteins were predicted to be involved in a remarkably wide variety of cellular processes. Finally, *ltp1*, encoding a novel lipid transporter and clustered with several TAG biosynthetic genes, was identified to play a role in lipid homeostasis of RHA1^19^.

Herein we describe whole transcriptomic analyses to elucidate the basis of N-mediated accumulation of TAGs in RHA1. Benzoate was used as a growth substrate because RHA1 grows well on this aromatic acid, similar to lignin depolymerization products, and catabolizes it via the β-ketoadipate pathway to yield acetyl-CoA and succinate^20^. The transcriptomic data were analyzed to further our understanding of how TAG biosynthesis is coordinated with metabolism in rhodococci. In addition, the role of a key *atf* gene was validated using targeted gene deletion. Phenotypic characterization included the determination of TAG content of whole cells and LDs. Finally, the studies identified a previously overlooked superfamily of enzymes that appear to be involved in TAG biosynthesis. The findings are discussed with respect to the engineering of rhodococcal strains for biotechnological applications.

## Results

### Accumulation of lipids in RHA1 under conditions of nitrogen limitation

To define N-limited conditions under which RHA1 accumulates TAGs, the strain was grown on 20 mM benzoate as sole growth substrate in M9 minimal medium, which contains 1 g l^−1^ ammonium chloride, or an otherwise chemically identical medium containing 0.05 g l^−1^ ammonium chloride. The cells had shorter doubling times on N-rich vs. N-limiting media, and grew to ~3-fold higher density in the N-rich medium (Fig. S1). The residual ammonium in the media at the time of harvesting the cells at early stationary phase was 0.73 g l^−1^ for N-rich and 0.001 g l^−1^ for N-limiting media. At these time points, the residual benzoate was undetectable (<80 μM) in N-rich media and 9.45 mM in N-limiting media. Based on these results, the two growth conditions were identified as N-excess and N-limited, respectively. The two cultures had similar pH values of 7.0 at early stationary phase. Finally, the growth yield (CDW) at the transition phase sampling time was 0.29 ± 0.03 mg ml^−1^ under conditions of N-limitation (72 h) and 0.78 ± 0.06 mg ml^−1^ under conditions of N-excess (91 h).

The neutral lipid content of whole cells was analyzed under N-excess and N-limited conditions at each of two time points: exponential growth and during the transition phase (*i.e*., from exponential growth to stationary phase). TLC analyses of total lipid extracts revealed that RHA1 accumulated significantly more TAGs in the N-limited condition during the transition phase (Fig. 2A). GC-MS analyses were performed to quantify the differences in TAG accumulation. During exponential growth under conditions of N-excess, FAs comprised <5% of the cells’ dry weight (Fig. 2B). This increased ~3-fold during the transition phase. By contrast, FAs comprised 12 ± 1% of the dry weight of exponentially growing cells under N-limited conditions, and this increased ~4.6-fold during the transition phase. This result was confirmed by Nile red staining (Fig. S3).

The GC-MS analyses further revealed that the FA profile depended on the growth phase of the cells. Thus, during exponential growth under N-limited conditions, over 80% of the FAs were of even-numbered chain length, with the major species being C16:0, C18:0 and C18:1 (Fig. 2C). By contrast, less than 60% of the FAs were of even-numbered chain length during the transition phase, with a significant decrease in the proportions of C18:0 and C18:1 species and an increase in the proportions of C15:0, C17:1, and especially C17:0 (~3-fold increase). The proportion of the most abundant FA, C16:0, was unchanged.

### Transcriptomic studies of RHA1 under different nitrogen conditions

To investigate the basis of TAG accumulation under N-limitation as well as how this biosynthesis is coordinated with cellular metabolism, we performed whole transcriptome analyses using RNA-seq. RHA1 was grown under N-excess and N-limited conditions, respectively, and the transcriptomes were analyzed at the same growth phases as the lipid analyses: exponential and transitional. To maximize rRNA depletion prior to cDNA library construction, a custom-designed depletion protocol using RHA1-specific 23*S* and 16*S* rRNA probes was developed. As summarized in Table S2, the Illumina sequencing provided between 283,000 and 1,263,000 reads that were used for RPKM calculations for each of the sampled conditions. A total of 2,527 genes were validated as being dysregulated in at least one of the comparisons between each of four experimental conditions (>2-fold change in abundance and p-value < 0.05; Table S3). In comparing the data sets, the greatest differences were observed between the two sets of data from the transition phase, with 1,826 genes dysregulated. Between the two data sets from exponentially growing cells, 779 genes were dysregulated. In cells growing under N-limited conditions, 482 genes had transcripts of different abundances during the transition phase versus exponential growth. In cells growing under conditions of N-excess, 1,324 genes were dysregulated between the two growth phases. In subsequent analyses, we focused on differences between cells in transition because (a) lipids accumulated to higher levels under these conditions and (b) the greatest differences in the transcriptomes were observed. The 1,826 genes that were dysregulated under these conditions include many involved in a variety of processes as summarized in Fig. 3, Table S4, and below.

Nitrogen metabolism – Genes known or predicted to be involved in N metabolism were among the most highly dysregulated (Table S4). Indeed, the transcripts of genes encoding two nitrite reductases (*nasDE* and *RHA1_RS31125-RHA1_RS31145*) were ~100- and 500-fold more abundant, respectively, during the transition phase of N-limited cells. Dysregulated genes potentially involved in assimilating ammonium included three encoding homologs of glutamate dehydrogenase (*RHA1_RS02795*, *RHA1_RS06755* and *RHA1_RS14810*) whose transcripts were less abundant and of two *glnA* homologs (*RHA1_RS05590* and *RHA1_RS13730*) whose transcripts were more abundant.

Growth substrate catabolism – Transcripts of the benzoate catabolic genes^20^ were also highly abundant during the transition phase due to N-limitation (Table S4). These include the genes encoding the uptake of benzoate and its transformation to catechol (*benABCDK*), the cleavage and transformation of the catechol (*catA1BC*) and the β-ketoadipate pathway (*pcaJ1I* and *pcaHBGLRF*) that yields acetyl-CoA and succinate. The RNA-seq data further revealed that these genes are organized into the 4 operons indicated in parentheses. The transcripts of several pathways that catabolize other growth substrates were less abundant under these conditions, such as phenylacetate (*paa*;)^21^. However, these had very low average RPKM values under conditions of N-limitation (Table S2).

**Figure 1:**
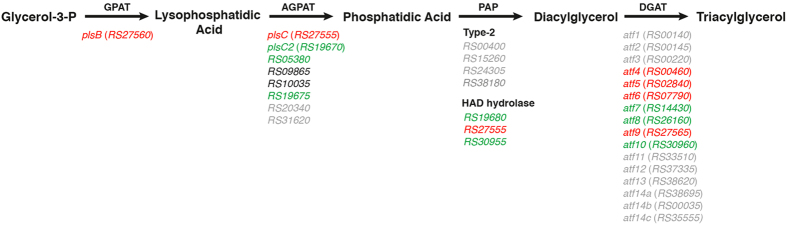
The Kennedy pathway of TAG biosynthesis. GPAT, glycerol-3-phosphate acyl transferase; AGPAT, acylglycerol-3-phosphate acyl transferase; PAP, phosphatidic acid phosphatase; DGAT, diacylglycerol acyl transferase. Homologous genes potentially encoding each step in RHA1 are marked as follows: green, transcripts more abundant during transition phase under N-limitation; red, transcripts less abundant during transition phase under N-limitation; grey, expressed at low levels under both conditions as defined by RPKM values that were less than 2.5% of total the RPKM value from all homologs predicted to catalyze that step. Type-2 PAPs and HAD-type hydrolases listed separately.

**Figure 2:**
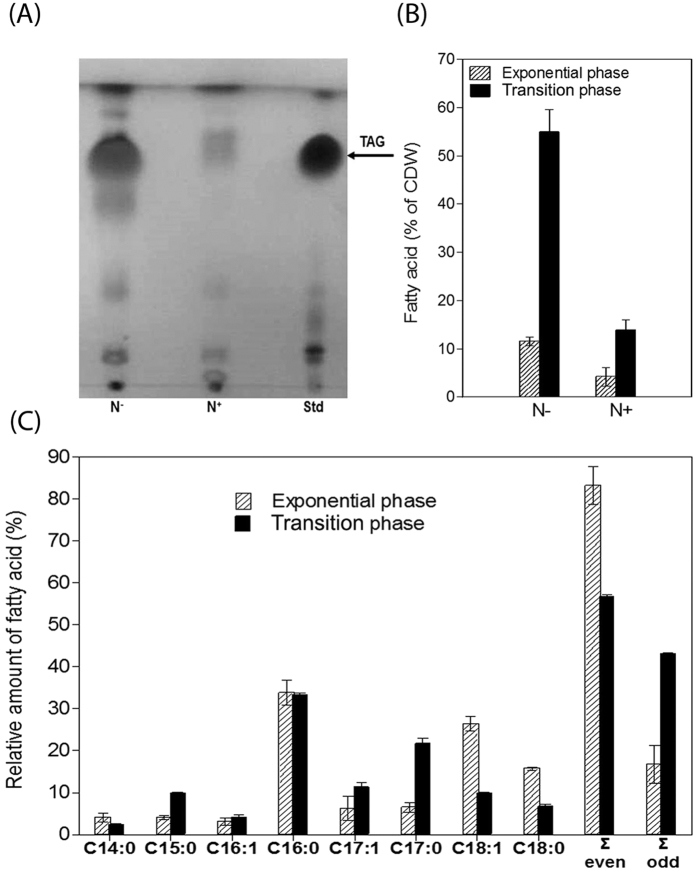
Lipid analysis of whole-cell extracts of benzoate-grown RHA1 under different nitrogen conditions. (**A**) TLC. Lanes: Std, TAG standard; N^−^, N-limiting condition; N^+^, N-excess condition during transition phase. (**B**) Total amounts of FAs (% of cellular dry weight) during exponential growth and transition phase under N-limiting (N^−^) or N-excess (N^+^) conditions. (**C**) FA composition of lipid accumulated by RHA1 under N-limitation during exponential growth and transition phase. Total FAs and relative amounts of each were measured by GC-MS. The data represent the means of three biological replicates.

Kennedy pathway – As summarized in Fig. 1 and Table S4, RHA1 contains multiple homologs of the Kennedy pathway enzymes, some of which have not been previously identified. Many of these genes are not clustered in the genome and their physiological roles have not been experimentally validated. A notable exception is *atf9*, *plsB* and *plsC*, predicted to encode each of the three acyltransferases of the Kennedy pathway. These genes were transcribed as an operon and *atf9* was the most abundant *atf* transcript under conditions of N-excess (Fig. 4). However, this transcript was 5.4-fold less abundant during transition phase under N-limitation (Table S4).

Of RHA1’s 16 *atf* homologs predicted to encode WS/DGATs, eight were dysregulated during the transition phase under conditions of different N availability. Those with the most abundant transcripts during N-limitation were *atf8* and *atf10* (Fig. 4A), which were respectively 49- and 6-fold more abundant under this condition (Table S4). By contrast, the most abundant *atf* transcripts during N-excess were *atf9*, *atf6* and *atf4*, which were 5.4-, 4.3- and 2.3-fold more abundant, respectively, under this condition. During exponential growth under conditions of N-excess, *atf6* and *atf9* were the most abundant *atf* transcripts. The respective operonic structures of these genes could be deduced from the RNA-seq data, except for that of *atf10* (Fig. 4B). The *atf10* operon has been provisionally identified to include six genes, including two that encode predicted FA desaturases (Table S4), and may be subject to regulation by an antisense RNA at the 3′ end of *RHA1_RS30965* (Fig. 4B). The *atf5*, *atf7* and *atf13* genes were also dysregulated during the transition phases, but their transcript levels were significantly lower overall.

The data provide interesting insight into RHA1’s other potential Kennedy pathway genes. Thus, of the eight genes predicted to encode AGPATs^17^,^19^ sharing 20–60% amino acid sequence identity, the transcripts of three were more abundant under N-limitation: *RHA1_RS05380*, *plsC2* and *RHA1_RS19675*. Of these, *RHA1_RS05380* was the most highly transcribed under N-limitation based on RPKM average values (Table S3), accounting for 50% of the AGPAT transcripts under these conditions. The RNA-seq data further indicate that *plsC2* and *RHA1_RS19675* occur in an operon with *RHA1_RS19680* (Fig. 4B), predicted to encode a haloacid dehalogenase (HAD)-type hydrolase. By contrast, no transcripts of genes encoding PlsB or PAP2 were more abundant under these conditions: transcripts of the lone *plsB* homolog were less abundant, as noted above, while transcripts encoding the four PAP2 homologs were present at very low levels.

**Figure 3:**
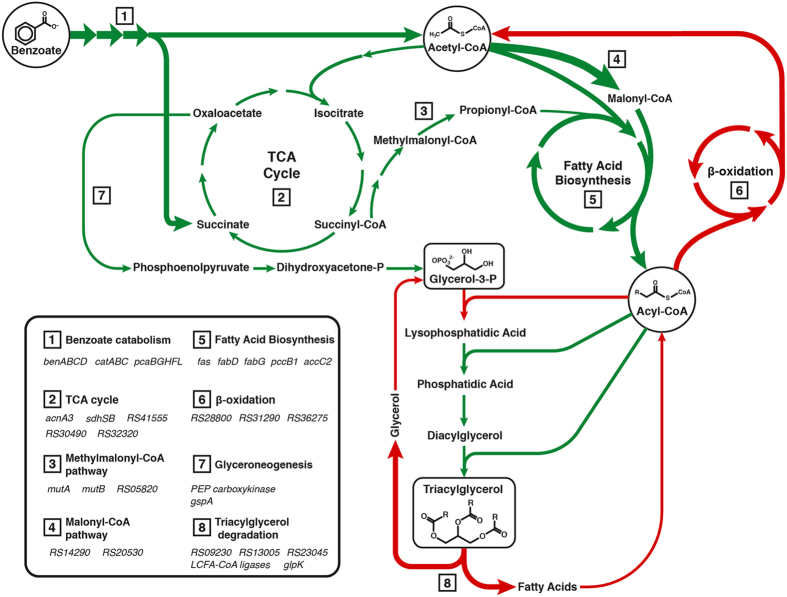
Overview of pathways related to TAG biosynthesis and transcript levels during the transition to stationary phase under N-limitation . Relevant genes are listed in the lower left chart. Arrows are colored as follows: green, transcripts of genes more abundant during N-limited transition; red, transcripts of genes less abundant during N-limited transition. Arrow thickness represents fold change: narrow, 2–10-times; regular, 10–100-times; wide, >100-times. Pyruvate was not explicitly included as a central metabolite for clarity. For the Kennedy pathway, the average RPKM values of the homologs listed in Fig. 1 were summed. For the TAG lipase step, the values of the three annotated homologs (listed as *RS* numbers under TAG degradation) were summed.

RHA1 genome contains at least 34 genes predicted to encode TAG lipases^2^, of which three are annotated as TAG lipases. The transcript of one of these, *RHA1_RS09230* was 16-fold less abundant during transition phase under N-limitation (Table S4) while those of the other two were at similar levels under the two conditions. Overall, the transcripts of the three TAG lipases were 10-fold less abundant (Fig. 3). Interestingly, the most strongly down-regulated gene in this data set, *RHA1_RS16675*, is annotated as an esterase/lipase (Table S4). However, this gene occurs in an operon encoding a predicted monooxygenase (*RHA1_RS16680*), suggesting that it is involved in a process other than TAG hydrolysis.

LD-associated proteins – Ding *et al.*^5^ identified 228 proteins associated with LDs in RHA1 while Chen *et al.*^16^ identified 177 in PD630. Transcripts associated with 103 of these were more abundant during the transition phase under N-limitation (Table S3). The dysregulated genes include *RHA1_RS10270*, corresponding to the LD-associated structure-like protein characterized by Ding *et al.*^5^ and TadA in PD630^18^.

Fatty acid biosynthesis and β-oxidation pathways – The RNA-seq data are consistent with increased synthesis of FAs and decreased FA β-oxidation during the transition phase of N-limitation. First, the transcripts of genes encoding two predicted acetyl-CoA carboxylases, *RHA1_RS20530* and *RHA1_RS14290*, were ~100-fold more abundant. These enzymes catalyze the first committed step in fatty acid biosynthesis by transforming acetyl-CoA to malonyl-CoA, an FA building block. Interestingly, *RHA1_RS14290* is co-transcribed with *RHA1_RS14295* which encodes a formyl-CoA transferase. Secondly, RHA1 harbors two FA synthases (FASs): FAS-I, encoded by *fas*, synthesizes FAs from C14 to C24 chain length in Actinobacteria^22^,^23^; and FAS-II, encoded by *acpS, fabD*, *acpP*-*fabF*, *fabG, pccB* and *accC2*, produces mycolic acids from the FAs generated by FAS-I^24^. Transcripts of *fas* were 115-fold more abundant while those of genes encoding FAS-II were up to 100-fold more abundant. Consistent with the synthesized FAs being incorporated into TAGs, the transcript of *RHA1_RS42105*, encoding a predicted long chain fatty acid CoA ligase, was 6-fold more abundant. Finally, with respect to β-oxidation, the transcripts of genes encoding acyl-CoA dehydrogenases (*RHA1_RS28800*, *RHA1_RS31290* and *RHA1_RS36275*) were less abundant.

Central metabolic pathways – Transcripts of genes encoding a variety of central metabolic pathways and enzymes were more abundant during the transition phase at N-limitation. These included those encoding the TCA cycle (14 transcriptional units, up to 39-fold more abundant), the methylmalonyl-CoA pathway (three transcriptional units, up to 10-fold), and the Entner-Doudoroff pathway (two transcriptional units, up to 14-fold). According to the KEGG database, RHA1 encodes 24 pyruvate dehydrogenases encoded by 14 different gene clusters (http://www.genome.jp/kegg/). Of these, five are operons whose transcripts were up to 39-fold more abundant under TAG accumulating conditions. Among central metabolic enzymes, TadD, an NAD(P)^+^-dependent glyceraldehyde 3-phosphate dehydrogenase, has been proposed to be a hallmark of TAG accumulation in PD630, effectively replacing glyceraldehyde 3-phosphate dehydrogenase (*gapA*) and phosphoglycerate kinase (*pgk*) which are present during vegetative growth^18^,^25^. This enables the cell to use NADPH under conditions of TAG accumulation instead of NADH and ATP. In RHA1 during TAG accumulation, the transcripts of *gap1* (*RHA1_RS16630*; formerly *ro03427*), *gap2* (*RHA1_RS35030*) and *pgk* (*RHA1_RS35035*) were 3-, 16- and 8-fold more abundant, respectively. Although there appears to be no direct homology of *tadD* in RHA1, three candidates were identified: *RHA1_RS29865*, *RHA1_RS27320* and *RHA1_RS30680*^17^. The RNA-seq data indicate that none of these genes were dysregulated under any of the studied conditions (Table S3).

Glyceroneogenesis – Glyceroneogenesis ensures the production of glycerol 3-phosphate, the starting point of the Kennedy pathway^17^,^26^. Two enzymes of glyceroneogenesis are pyruvate carboxylase, which catalyzes the formation of oxaloacetate from pyruvate, and phosphoenolpyruvate carboxykinase, which catalyzes the decarboxylation of oxaloacetate^26^. In RHA1, these are encoded by *pycA* (*RHA1_RS31870*) and *ppc* (*RHA1_RS35050*), respectively. Under N-limiting conditions, transcripts of *pycA* were almost 2-fold less abundant whereas *ppc* transcripts were 6-fold more abundant. The last step of glyceroneogenesis is catalyzed by NAD(P)H-dependent glycerol 3-phosphate dehydrogenase. Of the two genes predicted to encode this enzyme in RHA1, transcripts of *gpsA* (*RHA1_RS11710*) were 6-fold less abundant and those of *gpsA2* (*RHA1_RS31815*) were 3.4-fold more abundant.

### RT-qPCR analysis of RHA1 under nitrogen-limitation

The RNA-seq data of selected TAG biosynthesis genes were verified using RT-qPCR and the primers and probes listed in Table S1. Four *atf* genes in addition to *atf8* and *atf10* were included in this analysis: *atf3* and *atf6*, whose homologs in PD630 are physiologically important^13^,^15^; *atf9*, which is part of an operon with *plsC* and *plsB*; and *atf4*, whose product shares 47% amino acid sequence identity with Atf6. The RT-qPCR data largely validated the RNA-seq data although the exact values differed slightly (Table 1). Thus, only the transcripts *atf8* and *atf10* were more abundant during the transition phase under N-limitation (Table S4). By contrast, the transcripts of all the other tested *atf* genes were more abundant under conditions of N-excess, as were the *plsC* and *RHA1_RS16675* (putative esterase/lipase) transcripts. Finally, this experiment confirmed that expression of the gene encoding PAP-2 was low (data not shown) and did not appear to depend on N concentrations.

### Role of *atf8* on TAG accumulation in RHA1

To investigate the role of Atf8 in TAG biosynthesis, an *atf8* knockout mutant was constructed as summarized in Material and Methods. Growth of the mutant was comparable to that of the wild type under N-limited conditions (Fig. S1). TLC analysis of total lipids (Fig. 5A) showed a decrease in TAG content of the Δ*atf8* mutant compared to the wild type strain (WT) during N-limited transition phase. GC-MS analyses revealed that the mutant accumulated approximately one-third the amount of TAGs (w/w of the CDW) as the WT (Fig. 5B). The Δ*atf8* mutant was complemented using a replicative vector, pTip*atf8*, in which *atf8* is under control of a thiostrepton-inducible promoter. The production of Atf8 in the complemented mutant was verified using SDS-PAGE (Fig. S2). This revealed a protein of ~50 kDa, corresponding to the predicted molecular weight of Atf8, that was absent in the WT. Total lipid analysis showed that the complemented strain accumulated higher amounts of TAG in comparison with the Δ*atf8* mutant, but less than the WT cells (Fig. 5A,B). The partial complementation of Δ*atf8* may be due to the low solubility of Atf8 when over-produced in RHA1 (data not shown).

The pTip*atf8* construct was also used to investigate the effect of overproducing *atf8* in the WT strain. As in the complemented mutant, production of Atf8 was confirmed by SDS-PAGE (Fig. S2). Growth of the overproducing strain was comparable to that of WT under N-limitation (Fig. S1). TLC analyses of lipid content of the recombinant strain WT-pTip*atf8* revealed a slight increase in TAG content after the induction of *atf8* expression (Fig. 5A). Quantitative analyses revealed an increase of the total fatty acids of the WT-pTip*atf8* of ~20% (w/w of the CDW) as compared to the wild type (Fig. 5B). LDs were also purified from all RHA1 strains and lipid contents were analyzed by TLC (Fig. 6A). The qualitative differences in TAG recovered in the LDs of the RHA1 strains was consistent with what was observed in the total lipid extract, confirming the importance of *atf8* in TAG accumulation. Analysis of fatty acid composition revealed only slight differences between the WT and Δ*atf8* mutant strains (Fig. 5C). However, in strains overproducing Atf8, an increase of saturated fatty acids C15:0 and C17:0 and a decrease of unsaturated fatty acids C16:1, C17:1 and C18:1 was observed (Fig. 5C). The same pattern of fatty acid distribution was obtained in purified LDs (Fig. 6B).

Finally, to verify the activity of Atf8, DGAT activity was measured in the lysates of four strains of RHA1. In these experiments, lysates were prepared from cells at the transition phase under N-limitation, and diolein (DiC18:1) and palmitoyl-CoA (C16-CoA) were used as substrates. Lysate from WT RHA1 with the empty pTip-QC2 vector had a specific activity of 43 ± 3 nmol mg protein^−1^ min^−1^. By contrast, lysate of the strain containing pTip*atf8* contained 68 ± 9 nmol mg protein^−1^ min^−1^. Activities of the Δ*atf8* mutant carrying the pTip-QC2 and the pTip*atf8* vectors were 32 ± 5 and 47 ± 8 nmol mg protein^−1^ min^−1^, respectively. These data support the conclusion that *atf8* encodes a DGAT.

### An alternative PAP for the Kennedy pathway

**Figure 4:**
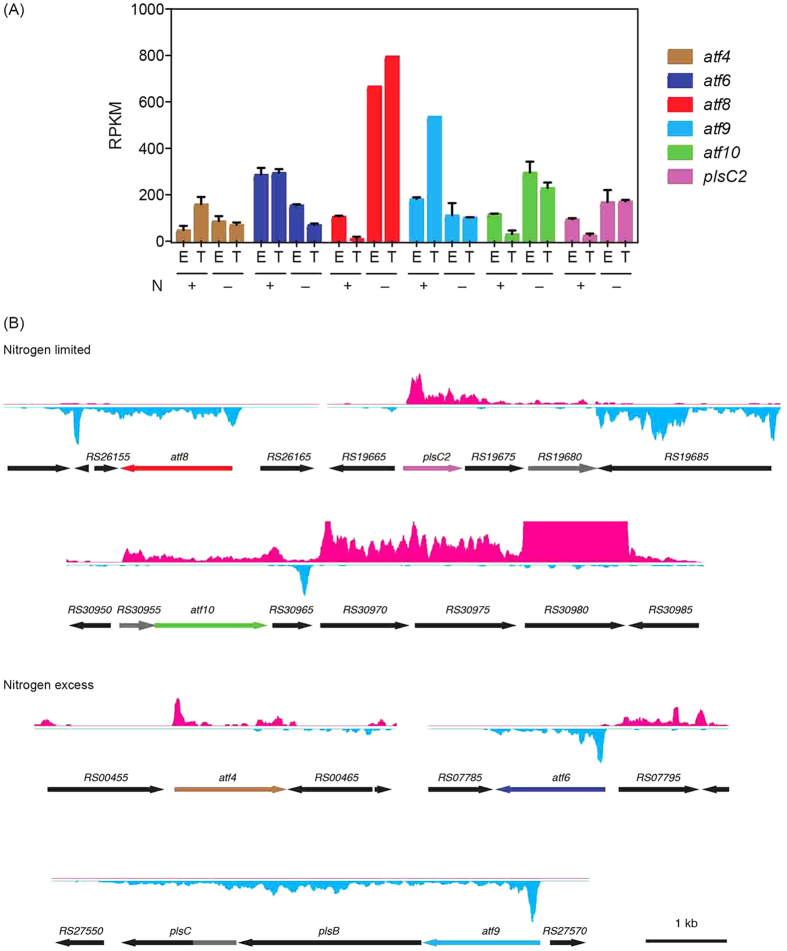
The expression and operonic organization of key TAG biosynthetic genes. (A) Transcript levels of *atf4*, *atf6*, *atf8*, *atf9*, *atf10* and *plsC* under each of the four experimental conditions. (**B**) Operon structure of the genes listed in panel (**A**).

**Figure 5:**
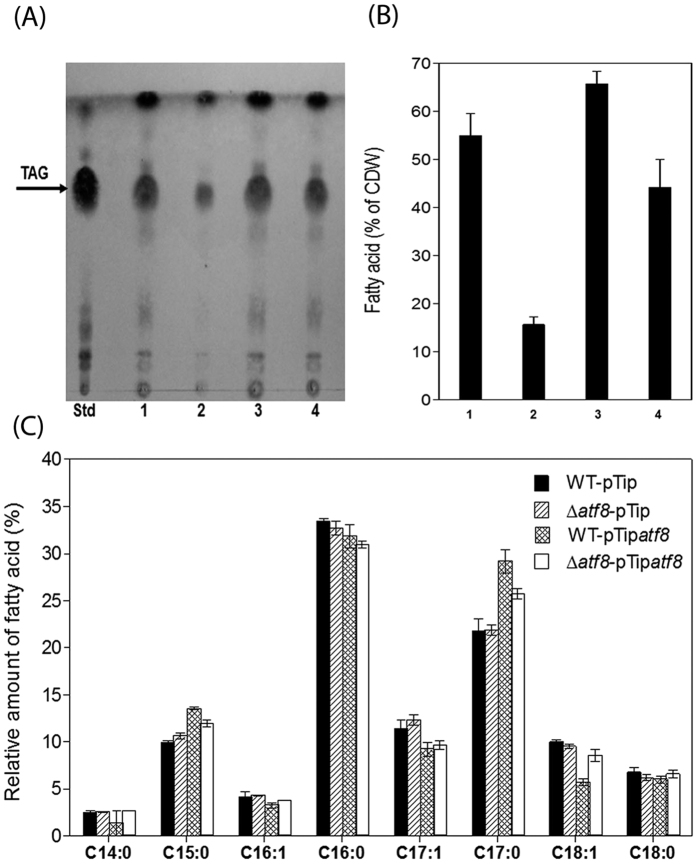
Lipid analysis of whole-cell extracts of benzoate-grown *atf8*-manipulated strains during transition phase under N-limitation. (**A**) TLC. Lanes: Std, TAG standard; 1, WT:pTip; 2, Δ*atf8*:pTip; 3, WT:pTip*atf8*; and 4, Δ*atf8*:pTip*atf8*. (**B**) Total amounts of fatty acids measured by GC-MS. Lanes numbered as in (**A**). (**C**) FA composition of RHA1 strains. Total amount of FA and the relative proportions of each type are presented as in Fig. 2. The data represent the means of three biological replicates.

**Figure 6:**
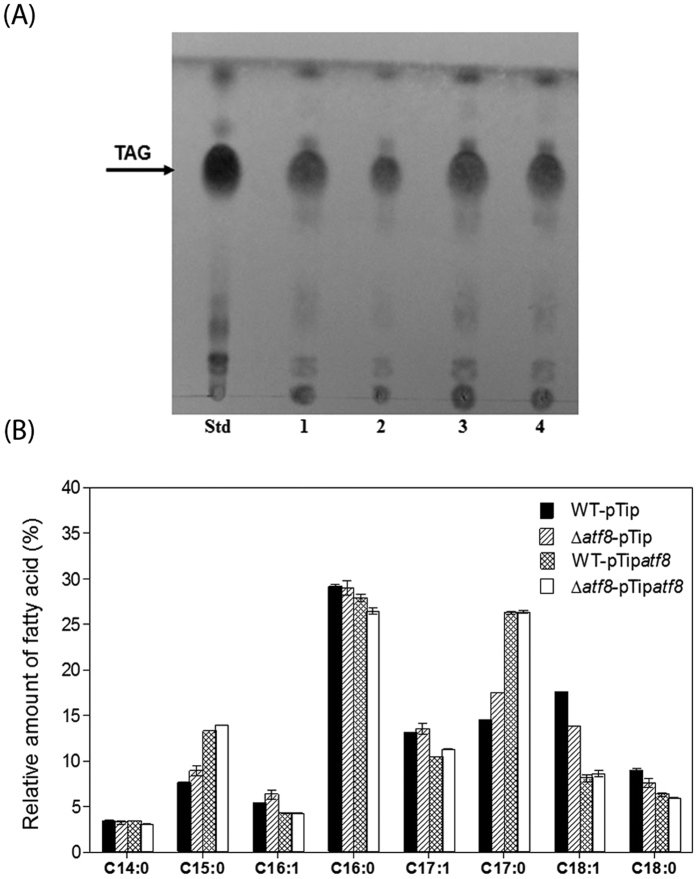
Analysis of lipid droplets from benzoate-grown RHA1 strains during transition phase under N-limitation. (**A**) TLC. Lanes: Std, TAG standard; 1, WT:pTip; 2, WT:pTip*atf8*; 3, Δ*atf8*:pTip; 4, Δ*atf8*:pTip*atf8*. (**B**) FA composition of RHA1 strains. The data represent the means of three biological replicates.

The low levels of the transcripts encoding the four PAP2 homologs prompted us to consider whether the third reaction of the Kennedy pathway might be catalyzed by another class of enzymes. Inspection of the operons encoding Kennedy pathway enzymes (Fig. 4B) revealed three genes predicted to encode HAD-type hydrolases according to NCBI: *RHA1_RS19680*, which occurs in the operon with *plsC2*; *RHA1_RS30955*, which is one of the genes of the potential *atf10* operon; and *plsC*, which appears to encode an AGPAT-hydrolase fusion enzyme. The encoded enzymes/domains share less than 20% sequence amino acid identity. Despite the name of the superfamily, many HAD-type hydrolases are phosphatases^27^.

## Discussion

Bacteria are of burgeoning interest in the development of consolidated bioprocesses for lignin^6^. RHA1 in particular has been identified as a promising starting point for metabolic engineering based on its ability to grow on a lignin-enriched stream derived from corn stover. In this study of TAG accumulation in RHA1, we used benzoate as a model of lignin-depolymerization products degraded via the β-ketoadipate pathway to succinate and acetyl-CoA. RHA1 accumulated significant amounts of TAG using this aromatic substrate under N-limitation. The transcriptomic and gene deletion experiments establish Atf8 as a key determinant of TAG accumulation under these conditions. More generally, this study advances our understanding of TAG metabolism in rhodococci, providing a framework for understanding how different homologs of the Kennedy pathway are utilized under different metabolic states of the cell. The data further indicate how metabolism directs carbon flow to TAG biosynthesis when growing on an aromatic compound, identify gaps in our knowledge of TAG biosynthesis, and provide some insight into other aspects of rhodococcal physiology.

TAG accumulation in benzoate-grown RHA1 was not as dramatic as what has been reported in rhodococci growing on other substrates and the identity of the FA species differed significantly. Previous studies of TAG biosynthesis in rhodococci have used glucose, gluconate or rich media as growth substrates^3^,^17^,^28^. FA contents as high as 75% and 85% CDW have been reported in PD630 grown on gluconate and olive oil, respectively^28^, higher than the maximum of 55% observed here for benzoate-grown RHA1 cells under conditions of N-limitation. It is unclear whether this reflects differences in the metabolism of these substrates. Consistent with previous reports of rhodococci grown on glucose and gluconate^28^, TAGs were the dominant neutral lipid. In contrast to the predominance of C16:0 and C18:1 FAs observed when glucose or gluconate was the growth substrate under N-limitation^2^,^17^, benzoate-grown RHA1 contained predominantly C16:0 (33%) and C17:0 (22%) FAs. Indeed, odd-numbered chain length FAs comprised more than 40% of the FAs under these conditions, indicating that propionyl-CoA was a significant building block for FAs. This building block could be generated from benzoate-derived succinate by the methylmalonyl-CoA pathway, which converts succinyl-CoA to propionyl-CoA in rhodococci^29^,^30^. Consistent with this explanation, this pathway was upregulated during TAG accumulation. The methylmalonyl-CoA pathway was also up-regulated during TAG accumulation in PD630^16^ and in gluconate-grown RHA1^17^. However, odd-numbered FAs comprised less than 30% of total FA in gluconate-grown RHA1, consistent with less propionyl-CoA being produced. Finally, the FA composition was also growth phase-dependent in the current studies, with exponentially growing cells containing ~2-fold greater proportion of C18:0 and C18:1 FAs and ~2-fold lesser proportion of C15:0 and C17:0 FAs. Overall, these results indicate that growth substrate and nutritional status influence the species of FA accumulated by *Rhodococcus*^28^,^31^,^32^,^33^.

The presented data identify Atf8 as the major DGAT responsible for the accumulation of TAGs under N-limitation. First, the *atf8* transcript was the most highly upregulated *atf* under these conditions and its transcript was 3.5-fold more abundant than any other. Second, deletion of *atf8* led to a 70% drop in TAG levels under N-limitation. By contrast, deletion of the *atf3* and *atf6* homologs in PD630 (*i.e*., *atf1*_PD630_ and *atf2*_PD630_) had a less dramatic effect, reducing TAG levels by 20% and 50%, respectively^13^,^15^. Importantly, heterologously produced Atf1_PD630_ had WS activity but not DGAT^13^. This is consistent with the low transcript levels of *atf3*. Consistent with our primary conclusion, Atf8 was also more abundant in RHA1 under lipid storage conditions in proteomic studies and was associated with LDs^17^. Moreover, the homolog of *atf8* in PD630, coincidentally *atf8*_PD630_, was also highly expressed during TAG accumulation^16^. Finally, the ability of the Δ*atf8* mutant to accumulate some TAG together with the transcriptomics data suggest that other DGATs such as Atf10 contribute to TAG biosynthesis during N-limitation. Again, this is consistent with previous reports of Atf10 being more abundant in RHA1 under lipid storage conditions^17^ and the corresponding gene being more highly expressed in PD630^16^. Nevertheless, Atf8 appears to be rate-limiting in TAG accumulation since its overexpression resulted in a 20% increase in accumulated TAG.

The data further implicate Atf6 and Atf9 as the primary DGATs responsible for TAG biosynthesis during exponential growth and that *atf6* and *atf9* are expressed during C-limitation. The regulation of *atf9* is particularly interesting since it occurs in the only operon predicted to encode all four activities of the Kennedy pathway as discussed below. Moreover, this operon is widely conserved in rhodococci and mycobacteria. Overall, the content, regulation and distribution of this operon suggest that it encodes “house-keeping” homologs of the Kennedy pathway. Interestingly, *ltp1* encoding a lipid transporter^19^ does not appear to be part of this operon. The lower levels of *plsB* transcript are somewhat unexpected since this is the only identified homolog in RHA1. Nevertheless, the transcriptomics results are largely consistent with published data obtained under different growth conditions. Thus, the homologs of Atf9 and PlsB in PD630 were repressed during TAG accumulation^16^, although direct comparison is difficult since the authors did not provide specific details on their growth conditions. Interestingly, Atf6 was more abundant in RHA1 under lipid storage conditions in proteomic studies^17^. Moreover, the corresponding enzyme in PD630, Atf2_PD630_, had significant DGAT activity in *E. coli* extracts^13^. It is possible that Atf6 is regulated at the transcriptional and translational levels.

The available evidence suggests the possibility that the three HAD-type hydrolases identified herein may be the major PAPs involved in TAG biosynthesis. First, all three are arranged in operons with genes encoding other enzymes of the Kennedy pathway; none of the genes encoding PAP2 homologs are. Second, transcripts of two of the HAD-type hydrolases were more abundant under TAG-accumulating conditions. By contrast, RHA1’s four genes encoding PAP2 homologs were present at very low levels in this study’s experimental conditions. Interestingly, the HAD-type hydrolases are conserved in the TAG gene clusters of mycolic acid-containing Actinobacteria such as rhodococci and mycobacteria (results not shown). This includes the *atf9*-*plsB*-*plsC* operon in which PlsC is a predicted fusion of AGPAT and HAD domains. The corresponding gene in *M. tuberculosis*, *Rv2483c*, was predicted to be required for growth in mice based on transposon mutagenesis^34^ but not for growth *in vitro*^35^. Clearly, additional work is required to establish the role of these HAD-type hydrolases. However, the possibility that PlsC is a bifunctional enzyme is particularly satisfying as this would mean that a single operon encodes all of the enzymes required for TAG biosynthesis during vegetative growth.

The transcriptomics data extend our understanding of how rhodococci shift their global metabolism to promote TAG accumulation. Pathways that generate propionyl-CoA and malonyl-CoA, the building blocks for FA biosynthesis, were among the most highly up-regulated including those involved in benzoate catabolism, the TCA cycle, the methylmalonyl-CoA pathway and acetyl-CoA carboxylases. Genes encoding the Entner-Doudoroff pathway and the pentose-phosphate pathway were also up-regulated. Increased flux through these pathways in gluconate-grown cells^17^ is not surprising considering that gluconate feeds into the Entner-Doudoroff pathway. The latter’s up-regulation during growth on benzoate suggests its primary importance in generating NADPH for FA biosynthesis. By contrast, NADPH does not appear to be generated by an NAD(P)^+^-dependent glyceraldehyde 3-phosphate dehydrogenase as occurs in PD630 under conditions of TAG accumulation^18^,^25^. Other up-regulated processes that provide building blocks for TAGs include glyceroneogeneis, fatty acid biosynthesis, and branched amino acid catabolism. These processes were also up-regulated under N-limiting TAG-accumulation during growth on gluconate^17^. Branched amino acid catabolism is a source of nitrogen, NADPH and TAG precursors. In glyceroneogenesis, the up-regulation of genes encoding PEP carboxykinase is consistent with the former catalyzing the rate-limiting step^26^. OAA for glyceroneogenesis may be generated through the TCA cycle as the lower abundance of *pycA* transcripts suggests that it is not generated through the carboxylation of pyruvate. Genes whose transcripts were less abundant include those involved in TAG degradation and FA oxidation. Interestingly, although eight predicted lipase-encoding genes were down-regulated during TAG accumulation, six were up-regulated (Table S4). Metabolic flux studies would be required to determine whether any turnover of TAGs occurs under these conditions.

Finally, the transcriptomics data highlight important gaps in our knowledge. Foremost, our knowledge of the Kennedy pathway in *Rhodococcus* is incomplete since, as noted above, the lone *plsB* homolog is down-regulated under TAG-accumulating conditions. At the same time, it is unclear why there is so much apparent redundancy in central metabolism in *Rhodococcus*. Most notably, genes encoding four predicted pyruvate dehydrogenases were highly up-regulated in this study. While this is consistent with a dramatic shift toward acetyl-CoA production during TAG accumulation, it is possible that some of these isozymes act on other α-keto acids. Similarly, two nitrite reductases, three putative glutamate dehydrogenases and two glutamine synthases (*glnA* and *glnA3*, a glutamate-ammonia ligase) were dysregulated. Although redundancy in rhodococcal catabolic enzymes is well known^20^, functional characterization of the central metabolic isozymes is required to establish their respective physiological roles.

We demonstrated that the *atf8*-encoded DGAT is a major determinant of TAG accumulation in RHA1 under N-limiting conditions. This DGAT likely plays the same role in PD630 and other oleaginous rhodococci in which it is conserved. Importantly, targeted disruption of *atf8* perturbed TAG accumulation to a greater extent than that reported in any other rhodococcal *atf* deletion to date. Moreover, overexpressing *atf8* significantly increased the production of TAG in benzoate-grown RHA1, establishing the potential of engineering rhodococci to optimize TAG production from different substrates. Finally, we identified a previously overlooked suite of HAD-type hydrolases that appear to be involved in TAG biosynthesis. These findings, together with the transcriptomics data, should facilitate the engineering of rhodococci for the production of TAGs as feedstock for various applications^9^,^10^.

## Methods

### Strains and culture conditions

*E. coli* DH5α was used for DNA propagation and manipulation. *E. coli* strains were grown at 37 °C (200 rpm) in LB broth unless otherwise indicated. Bacto agar (1.5% [w/v]; Difco) was used for solid medium. RHA1 and derivative strains were cultivated at 30 °C under aerobic conditions in M9 minimal medium supplemented with trace elements, thiamin and 20 mM benzoate as growth substrate in shake flasks. For nitrogen-limiting conditions, the concentration of ammonium chloride in the M9 medium was reduced from 1 g l^−1^ to 0.05 g l^−1^. Expression from the thiostrepton-inducible promoter (P_*tipA*_) of pTip-QC2 was induced by adding 10 μg ml^−1^ of thiostrepton to the culture. Growth was measured as the change in optical density at 600 nm. For lipid analyses, cells were harvested, washed with 150 mM NaCl and frozen at −80 °C. As appropriate, antibiotics were used at the following final concentrations: 100 μg ml^−1^ ampicillin, 50 μg ml^−1^ kanamycin and 34 μg ml^−1^ chloramphenicol.

### DNA manipulation

DNA was manipulated using standard protocols^36^. Oligonucleotides were from Integrated DNA Technologies. The sequences of primers and probes used in this study are listed in Table S1. DNA sequencing was performed at Genewiz Inc. *E. coli* and RHA1 were transformed with plasmid DNA by electroporation using a MicroPulser with GenePulser cuvettes (Bio-Rad). The Δ*atf8* mutant was constructed using the *sacB* counter selection system^37^. The flanking regions of *atf8* were amplified from RHA1 genomic DNA using the *atf8*-up and *atf8*-dn oligonucleotides (Table S1). The two amplicons of ~500 bp and were cloned into pK18mobsacB using restriction enzymes *Xba*I, *Bam*HI and *Hin*dIII to yield pK18MSatf8. Kanamycin-sensitive/sucrose-resistant colonies were screened using PCR. The nucleotide sequence of the targeted region was determined in the mutant strain to confirm the gene deletion. For complementation and over-expression experiments, *atf8* was amplified from RHA1 genomic DNA using primers *atf8*-ex-F and *atf8*-ex-R (Table S1) together with GoTaq DNA polymerase (Promega). The resulting amplicon was digested with *Nde*I and *Eco*RI and cloned in pTip-QC2^38^,^39^ yielding pTip*atf8*. The nucleotide sequence of the cloned gene was verified and the vector was used to complement the mutant and to overexpress *atf8* in WT RHA1.

### RNA isolation

Total RNA was extracted using TRIzol^®^ Reagent (Invitrogen) according to the manufacturer’s instructions. The RNA was treated with TURBO^TM^ DNase (Invitrogen) and extracted with phenol-chloroform. The quality and concentration of RNA were assessed using a 2100 Bioanalyzer and the RNA 6000 Nano kit (Agilent). Three μg of total RNA was heated at 65 °C for 5 min, quickly chilled on ice and then mixed with Streptavidin magnetic beads (New England Biolabs) with an equimolar mixture of biotin-labelled probes specific for RHA1 16*S* and 23*S* rRNA to deplete rRNA (rRNA-16*S*, rRNA-23*S*a, and rRNA-23*S*b (Table S1)). The supernatant was collected and subjected to further rRNA-depletion using the Ribominus™ Transcriptome Isolation Kit (Invitrogen).

### RNA-seq analysis

One hundred ng of the rRNA-depleted RNA was used for library construction using NEBNext^®^ Ultra Directional RNA Library Prep Kit for Illumina^®^ and NEBNext^®^ Multiplex Oligos for Illumina^®^ (New England Biolabs) according to the manufacturer’s instructions. The samples were analyzed using a Genome Analyzer IIx (Illumina^®^). Data were processed using the Sequence Control Software (Illumina^®^). Single-ended sequence reads were scanned 3′ to 5′. Those with a Phred quality value below 20 were trimmed using the FASTQ Quality Trimmer tool of the FASTX-toolkit (http://hannonlab.cshl.edu/fastx_toolkit/index.html). Reads of less than 35 bases after trimming were discarded from further analysis. Trimmed reads were aligned to the RHA1 genome^40^ using Bowtie 2 in end-to-end mode with the “very sensitive” option^41^. Reads of rDNA sequences were omitted in subsequent analyses. A custom Perl script was developed to create a GTF file using the recently updated RHA1 annotation by the NCBI Prokaryotic Genome Annotation Pipeline. Mapped read counts per gene were obtained using the HTSeq package^42^. Differential expression (>2-fold change with a false discovery rate-adjusted p value < 0.05) was analyzed using the *DESeq2* package^43^. The RNA-seq data set was deposited in Gene Expression Omnibus^44^ under accession number of GSE77158.

### RT-qPCR

Primers and FAM-labelled TaqMan probes (Table S1) were designed using IDT PrimeTime qPCR design tools. The reference gene was *sigA* (*RHA1_RS33345*), which encodes the major sigma factor in RHA1. cDNA was synthesized from DNase-treated, Trizol-extracted total RNA using the SuperScript® VILO cDNA synthesis kit according to manufacturer instructions. The cDNA samples were diluted as appropriate for use. Reactions were performed in duplicate using an ABI stepOnePlus real-time PCR system and the following conditions: 20 s at 95 °C followed by 40 cycles of 1 s at 95 °C and 20 s at 60 °C for extension. Standard curves for each target based on genomic DNA were included in each run to quantify absolute target cDNA in the samples. Concentrations were normalized to those of the reference gene under each condition tested. Relative fold differences were calculated using conditions of nitrogen excess as a reference.

### Isolation of LDs

LDs were isolated essentially as previously reported^5^. Briefly, RHA1 cells were cultivated in shake flasks in 50 ml M9 medium, harvested by centrifugation (4,000 × g for 10 min) and washed twice with 20 ml Buffer A (25 mM tricine, 250 mM sucrose, pH 7.8). The washed cells were suspended in 20 ml Buffer A, incubated for 20 min on ice and disrupted by passing them three times through a French pressure cell operated at 100 MPa, 4 °C. The sample was centrifuged at 4,000 × *g* for 10 min to remove cell debris. Eight ml of the supernatant was loaded into an SW40 tube (Beckman), layered with 2 ml Buffer B (20 mM HEPES, 100 mM KCl, 2 mM MgCl_2_, pH 7.4) on top, and centrifuged at 180,000 × *g* for 1 h at 4 °C. The LD fraction on top of the sucrose gradient was collected and transferred to a 1.5 ml Eppendorf tube. LDs were washed with 200 μl Buffer B. To dissolve the lipids and to precipitate proteins, 1 ml chloroform-acetone (1:1, v/v) was added. The sample was vortexed and centrifuged (20,000 g × 10 min). Finally, the organic phase was collected, dried under a nitrogen steam, and suspended in 100 μl chloroform for lipid analysis.

### Lipid extraction and thin-layer chromatography (TLC)

For whole RHA1 lipid content analysis, cells were harvested during the transition from exponential to stationary growth phases, washed with a 150 mM NaCl and suspended in 50 mM Tris-HCl, pH 7.0, and 300 mM NaCl buffer. Cells were subjected to four rounds of 60 s of bead beating using a FastPrep-24 bead beater (MP Biomedicals, Solon, OH) set to 5.5. The sample was incubated for 5 min on ice between rounds. To identify lipids, samples of whole cells or isolated LDs were extracted with chloroform-methanol (2:1, v/v). Aliquots were analyzed by thin layer chromatography (TLC) on 60F254 silica gel plates (Merck) using hexane/diethyl ether/acetic acid (80:20:1, v/v/v) as a solvent system. Triolein (Sigma-Aldrich, T7140) was used as the TAG standard. The plates were dried at room temperature for 10 min and immediately sprayed with a 10% cupric sulfate in 8% phosphoric acid solution. The plates were then incubated in an oven at 150 °C for 10 min.

### Fatty acid profiling

FA profiles were determined by subjecting 5 mg of freeze-dried whole cells or LD extracts to methanolysis in the presence of 15% (v/v) sulfuric acid as described by Alvarez *et al.*^28^. The resulting methylesters were analyzed using an Agilent 6890 series gas chromatograph (GC) equipped with an HP-5 MS 30 m × 0.25 mm capillary column (Hewlett-Packard) and an HP 5973 mass-selective detector. The GC was operated with an injector temperature of 280 °C, a transfer line temperature of 290 °C, and a flow rate of 1 ml min^−1^ with helium. The temperature program of the oven was 90 °C for 5 min, increased to 240 °C at a rate of 6 °C per min, and then held at 240 °C for 17 min. The mass spectrometer was operated in electron emission scanning mode at 40 to 800 *m*/*z* and 1.97 scans per second. Peaks were quantified and identified using the software packages GCsolution Analysis version 2.32 and GCMS Solutions version 2.53 (Shimadzu Scientific Instruments, Columbia, MD) and the NIST08 Library.

### Ammonium determination

Ammonium concentration was quantified in culture media using indophenol blue^45^. Briefly, media samples were diluted 50–1000-fold to 10 ml using distilled water. To this were added: 0.4 ml of reagent (10% w/v phenol solution in ethanol), 0.4 ml catalyst (0.5% w/v nitroprusside in water) and 1 ml oxidizing solution (20% w/v trisodium citrate and 1% w/v sodium hydroxide solution, mixed with 5% sodium hypochlorite 4:1 v/v). Samples were incubated at 37 °C with shaking for 2 h in the dark. Absorbance was read at 630 nm and concentrations were calculated using a standard curve of 0.2–2 mg ml^−1^ ammonium chloride.

### Protein production, purification and analysis

Protein extracts were prepared from RHA1 by suspending cells in 50 mM Tris-HCl, pH 7.0 containing 300 mM NaCl and 1 mg ml^−1^ CHAPS. Cells were subjected to four rounds of 60 s of bead beating as described above. Protein extracts were analyzed by SDS-PAGE.

### DGAT activity

DGAT activity was measured using a spectrophotometric assay that monitors the reaction of dithionitrobenzoate (DTNB) with the CoASH liberated from the DGAT-catalyzed esterification of acyl-CoA and diglyceride^4^. Briefly, assays were performed in a total volume of 1 ml and absorbance was recorded at 412 nm. Ten μl DTNB solution (18 mg ml^−1^ in dimethyl sulfoxide) and 15 μl each of 1 mM palmitoyl-CoA and 1 mM diolein were added to 950 μl 50 mM Tris-HCl, 150 mM NaCl, pH 8.0. The base line was recorded, the reaction was initiated with 10 μl of lysate, and the absorbance was recorded for 1 min. Initial reaction rates were calculated from the data using Excel and an ε_412_ of 14,150 M^−1^cm^−1^ for the nitrothiobenzoate dianion.

### Computational analysis

Database searches and alignments were carried out using BLAST 2.2.17^46^ and CLUSTALW^47^. Reference protein sequences were retrieved from the NCBI database.

## Additional Information

**How to cite this article**: Amara, S. *et al.* Characterization of key triacylglycerol biosynthesis processes in rhodococci. *Sci. Rep.* **6**, 24985; doi: 10.1038/srep24985 (2016).

## Supplementary Material

Supplementary Information

Supplementary Dataset

## Figures and Tables

**Table 1:** RT-qPCR analysis of selected TAG metabolic genes in RHA1 during transition phase under different N conditions.

Gene ID^a^	Gene Name	Product	Fold change
*RS00220*	*atf3*	WS/DGAT	−3
*RS00400*		PAP-2	1
*RS00460*	*atf4*	WS/DGAT	−5
*RS07790*	*atf6*	WS/DGAT	−4
*RS16675*		putative lipase	−1596
*RS26160*	*atf8*	WS/DGAT	28
*RS27555*	*plsC*	AGPAT	−50
*RS27565*	*atf9*	WS/DGAT	−35
*RS30960*	*atf10*	WS/DGAT	3

^a^*RHA1_* prefix has been omitted for simplicity.
