# Supplementary material for: Characterization of key triacylglycerol biosynthesis processes in rhodococci
Source: Sci Rep. 2016 Apr 29;6:24985. doi: 10.1038/srep24985 (PMC4850399; doi:10.1038/srep24985)
Supplement: Supplementary Information [file srep24985-s1.doc]

**Characterization of key triacylglycerol biosynthesis processes in rhodococci**

Sawsan Amara^1^, Nicolas Seghezzi^1^, Hiroshi Otani^1^, Carlos Diaz-Salazar^1^, Jie Liu^1^ and Lindsay D. Eltis^1*^

.

^1^ Department of Microbiology and Immunology, Life Sciences Institute, The University of British Columbia, 2350 Health Sciences Mall, Vancouver, BC V6T 1Z3, Canada

^*^Corresponding author.

Tel: +1 604 822-0042; Fax: +1 604 822-6041; E-mail: leltis@mail.ubc.ca

**Supplementary Methods**

**Lipid staining with Nile red**. Nile red (NR, Sigma-Aldrich) was prepared as a stock solution of 1 mg ml^-1^ in DMSO. Staining was performed as previously described^1^. Briefly, RHA1 cultures (~10^7^ cells ml^-1^) where suspended in 150 mM NaCl solution and stained with NR (1 µg ml^-1^) followed by 10 min of incubation in darkness before measuring the fluorescence intensity of NR using Varioskan Flash apparatus (Thermo Scientific).

**Supplementary References**

1 Chen, W., Zhang, C., Song, L., Sommerfeld, M. & Hu, Q. A high throughput Nile red method for quantitative measurement of neutral lipids in microalgae. *J Microbiol Methods* **77**, 41-47 (2009).

**Supplementary Figures**

**Figure S1.** Growth of *R. jostii* RHA1 in M9 medium under N-limitation (full symbols) or N-excess (empty symbols) using benzoate as sole carbon source. Arrows represent sampling points for transcriptomic and lipid analysis during exponential or stationary growth phases.

**WT-pTip N^-^**

**WT-pTip*atf8* N^-^**

**     Δ*atf8*-pTip N^-^**

**     Δ*atf8*-pTip*atf8* N^+^**

**WT-pTip N^+^**

**WT-pTip*atf8* N^+^**

**     Δ*atf8*-pTip N^+^**

**     Δ*atf8*-pTip*atf8* N^+^**

**Figure S2.** SDS-PAGE gel of various RHA1 strains. Cells were harvested 24h after thiostrepton induction. Total lysate was then analyzed by SDS-PAGE. Lanes: MT, molecular weight standard; 1, WT-pTip; 2, WT-pTip*atf8*; 3, Δ*atf8*-pTip; 4, Δ*atf8*-pTip*atf8*. Arrows indicate Atf8.


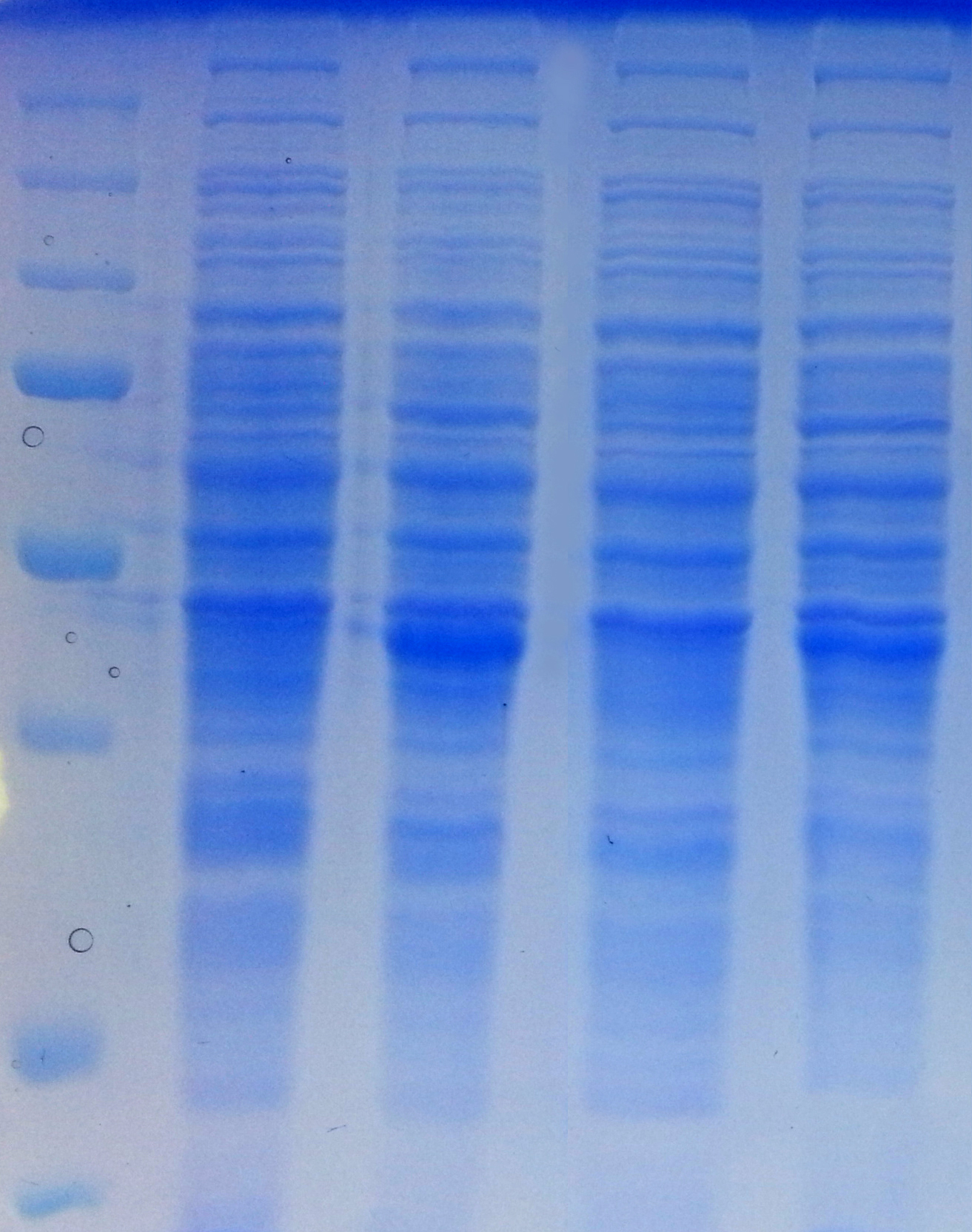


**50**

**37**

**MT 1 2 3 4**

**Figure S3**. NR staining of benzoate-grown RHA1 during transition phase under N-limiting (N-) and N-excess (N+) conditions.

**Supplementary Tables**

**Table S1.** Oligonucleotides used in this study.

| Oligo name^a^ | Nucleotide sequence | |  |
| --- | --- | --- | --- |
| Depletion of *rRNA* for RNA-seq experiments^b^ | |  | |
| rRNA16*S* | ATATGTCAAACCCAGGTAAGGTTCTTC | |  |
| rRNA23*S*a | AGGAATTTCGCTACCTTAGGATGGTTATAGTTACC | |  |
| rRNA23*S*b | CTTTTCACCTTTCCCTCACGGTACTAGTCCGCTAT | |  |
| RT-qPCR | |  | |
| *atf3*-F | GATTCCGTTTTCCCCGATGA | |  |
| *atf3*-R | GTGTTTGAGCATGTTTCCGAG | |  |
| *atf3*-probe | CCTGCTTCCCGCCTCGACA | |  |
| *RS00400*-F | TGGTGATCGTTGCCGTGA | |  |
| *RS00400*-R | CGCCTACCGTCACGAACA | |  |
| *RS00400*-probe | CAGTGGACACCGAGGTAGACGC | |  |
| *atf4*-F | TGATGATCTCGTACATGCCG | |  |
| *atf4*-R | GTGAGGGTGATGCTGAGTG | |  |
| *atf4*-probe | AGGCCGCGATACTGGAATGGG | |  |
| *atf6*-F | GAAGACCATGTACTGGAACGG | |  |
| *atf6*-R | TGGTGAGCGTGATGTTGAG | |  |
| *atf6*-probe | CGGCTGGACGGCATCTACCC | |  |
| *RS16675*-F | TGATCTACCCGGTGATCGAT | |  |
| *RS16675*-R | GAGGACAGGTAGTTGCTCCA | |  |
| *RS16675*-probe | CGTACCCCTCGGCGAACTCC | |  |
| *atf8*-F | GGAGCTGATGGCACTCTG | |  |
| *atf8*-R | GTGGATCTTGGTGTAGACGG | |  |
| *atf8*-probe | CTGATCGAGGGACTGGGCGAC | |  |
| *plsC*-F | ATCTGCGGGATCAACGTG | |  |
| *plsC*-R | AGCGAACTCTGGTGATTGAAC | |  |
| *plsC-*probe | CGCATCAAGGGCAAGGAGAACG | |  |
| *atf9*-F | ACATGGTGCCGCGTATC | |  |
| *atf9*-R | GACAAGTGATAGTCGAGGTCG | |  |
| *atf9*-probe | CCACATCGGATTGCCCAGCC | |  |
| *atf10*-F | AGCGGATGTTCAACCTGATG | |  |
| *atf10*-R | ACCGATGCTCAAAGTCTGG | |  |
| *atf10-*probe | AGCGCGGATGCTCGAGATGT | |  |
| *sigA*-F | CTGATCCAGGAAGGCAACC | |  |
| *sigA*-R | AGAACTTGTAGCCCTTGGTG | |  |
| *sigA*-probe | CGAACTTCTCGACGGCACGGAT | |  |
| Construction of *Δatf8* mutant | |  | |
| *atf8*-up-F | GCTCTAGACGGCAGTTCGGACGCTCGGTAC^c^ | |  |
| *atf8*-up-R | TTGGATCCGGACTCGCCGAGAAGGAACATCG | |  |
| *atf8*-dn-F | TTGGATCCGTGCCCGACCTGAAGTCGATCC | |  |
| *atf8*-dn-R | TTTAAGCTTGACGGCCTGGATCTTCTCGTTGG | |  |
| Expression of *atf8* and complementation of *Δatf8* mutant | |  | |
| *atf8*-ex-F | ACGAGCCATATGCCGCTCCCCATGTCCCC | |  |
| *atf8*-ex-R | ACGAGCGAATTCTCAGATTCCGACCGCGCGCT | |  |

^a^Name begins with gene name or *RHA1_* gene ID.

^b^Experiment in which oligonucleotide was used.

^c^Restriction site underlined.

**Table S2.** RNA-seq data for RHA1 cells grown under conditions of N-limitation and N-excess, sampled at mid-exponential and transition phases.

| Experimental condition | Mapped reads | rRNA reads | Reads used for RPKM |
| --- | --- | --- | --- |
| N-excess, exponential-1 | 3,724,008 | 3,440,460 | 283,548 |
| N-excess, exponential-2 | 4,352,693 | 3,951,834 | 400,859 |
| N-excess, transition-1 | 3,729,680 | 3,344,280 | 385,400 |
| N-excess, transition-2 | 1,154,626 | 1,015,868 | 138,758 |
| N-limited, exponential-1 | 1,526,358 | 1,388,034 | 138,324 |
| N-limited, exponential-2 | 4,474,278 | 4,114,279 | 359,999 |
| N-limited, transition-1 | 5,802,317 | 4,904,665 | 897,652 |
| N-limited, transition-2 | 8,542,433 | 7,279,127 | 1,263,306 |

**Table S3.** All dysregulated genes.

See Excel file.

**Table S4. Differential expression of predicted key TAG/WE metabolic genes under conditions of N-limitation versus N-excess in early stationary phase.a**

| Gene ID  (RHA1_) | Gene name | Product | Fold change |
| --- | --- | --- | --- |
| **Kennedy pathway** | | | |
| *RHA1_RS00460* | *atf4* | diacylglycerol *O*-acyltransferase | -2.3 |
| *RHA1_RS02840* | *atf5* | diacylglycerol *O*-acyltransferase | -3.3 |
| *RHA1_RS05375* |  | membrane protein | 4.9 |
| *RHA1_RS05380* |  | 1-acyl-sn-glycerol-3-phosphate acyltransferase | 3.6 |
| *RHA1_RS05385* |  | glucokinase | -4.2 |
| *RHA1_RS07790* | *atf6* | diacylglycerol *O*-acyltransferase | -4.3 |
| *RHA1_RS14430* | *atf7* | diacylglycerol *O*-acyltransferase | 4.5 |
| *RHA1_RS19670* | *plsC2* | 1-acylglycerol-3-phosphate acyltransferase | 5.6 |
| *RHA1_RS19675* |  | 1-acylglycerol-3-phosphate acyltransferase | 2.8 |
| *RHA1_RS19680* |  | HAD family hydrolase | 3.7 |
| *RHA1_RS26160* | *atf8* | diacylglycerol *O*-acyltransferase | 49 |
| *RHA1_RS27555* | *plsC* | 1-acylglycerol-3-phosphate *O*-acyltransferase | -4.4 |
| *RHA1_RS27560* | *plsB* | glycerol-3-phosphate acyltransferase | -6.2 |
| *RHA1_RS27565* | *atf9* | diacylglycerol *O*-acyltransferases | -5.4 |
| *RHA1_RS30955* |  | HAD family hydrolase | 7.6 |
| *RHA1_RS30960* | *atf10* | diacylglycerol *O*-acyltransferase | 6.0 |
| *RHA1_RS30965* |  | hypothetical protein | 1.1 |
| *RHA1_RS30970* |  | stearoyl-CoA 9-desaturase | 2.2 |
| *RHA1_RS30975* |  | fatty acid desaturase | 2.6 |
| *RHA1_RS30980* |  | fatty acid desaturase | 6.8 |
| *RHA1_RS33510* | *atf13* | diacylglycerol *O*-acyltransferase | 8.2 |
| **Nitrogen metabolism** | | | |
| *RHA1_RS02795* | *gdhA* | amino acid dehydrogenase | -3.7 |
| *RHA1_RS04115* | *nasD* | nitrite reductase | 120 |
| *RHA1_RS04120* | *nasE* | 2Fe-2S ferredoxin | 112 |
| *RHA1_ro01157* | *glnA1* | glutamine synthetase | 8.0 |
| *RHA1_RS06745* |  | hypothetical protein | -1.4 |
| *RHA1_RS06750* |  | 4-hydroxybenzoyl-CoA thioesterase | -1.8 |
| *RHA1_RS06755* |  | NAD^+^-glutamate dehydrogenase | -2.7 |
| *RHA1_RS13710* |  | allantoate amidohydrolase | 22.4 |
| *RHA1_RS13715* |  | glutamate synthase α subunit | 30.0 |
| *RHA1_RS13720* |  | protein GlxC | 21.1 |
| *RHA1_RS13751* |  | glutamine amidotransferase-like protein | 14.0 |
| *RHA1_RS13730* | *glnA3* | glutamate-ammonia ligase | 30.3 |
| *RHA1_RS13735* |  | FAD-dependent oxidoreductase | 15.5 |
| *RHA1_RS14810* | *gdhA2* | amino acid dehydrogenase | -17.4 |
| *RHA1_RS14815* |  | amino acid transporter | -54.2 |
| *RHA1_RS31125* | *narK* | major facilitator transporter | 295 |
| *RHA1_RS31130* |  | nitrite reductase | 535 |
| *RHA1_RS31135* |  | nitrite reductase | 154 |
| *RHA1_RS31140* |  | uroporphyrinogen III synthetase | 84.4 |
| *RHA1_RS31145* |  | cobalamin biosynthesis protein CbiX | 74.7 |
| **Benzoate catabolism** | | | |
| *RHA1_RS06455* | *pcaJ1* | succinyl-CoA:3-ketoacid-CoA transferase | 43.5 |
| *RHA1_RS06460* | *pcaI* | succinyl-CoA:3-ketoacid-CoA transferase | 21.1 |
| *RHA1_RS06465* | *pcaH* | protocatechuate 3,4-dioxygenase, β subunit | 29.1 |
| *RHA1_RS06470* | *pcaG* | protocatechuate 3,4-dioxygenase, α subunit | 30.8 |
| *RHA1_RS06475* | *pcaB* | 3-carboxy-*cis*,*cis*-muconate cycloisomerase | 86.4 |
| *RHA1_RS06480* | *pcaL* | 3-oxoadipate enol-lactone hydrolase | 122 |
| *RHA1_RS06485* | *pcaR* | IclR family transcriptional regulator | 73.5 |
| *RHA1_RS06490* | *pcaF* | acetyl-CoA acetyltransferase | 47.7 |
| *RHA1_RS11585* | *catC* | muconolactone delta-isomerase | 302 |
| *RHA1_RS11590* | *catB* | chloromuconate cycloisomerase | 335 |
| *RHA1_RS11595* | *catA1* | catechol 1,2-dioxygenase | 208 |
| *RHA1_RS11600* | *catR* | IclR family transcriptional regulator | 18.2 |
| *RHA1_RS11650* | *benA* | benzoate 1,2-dioxygenase, α subunit | 145 |
| *RHA1_RS11655* | *benB* | benzoate 1,2-dioxygenase, β subunit | 196 |
| *RHA1_RS11660* | *benC* | benzoate 1,2-dioxygenase ferredoxin reductase | 254 |
| *RHA1_RS11665* | *benD* | 1,6-dihydroxycyclohexa-2,4-diene-1-carboxylate dehydrogenase | 328 |
| *RHA1_RS11670* | *benK* | MFS transporter | 96.4 |
| **Lipases** | | | |
| *RHA1_RS05030* |  | lipase | -3.4 |
| *RHA1_RS06260* |  | hypothetical protein | 6.6 |
| *RHA1_RS06265* |  | lysophospholipase | 12 |
| *RHA1_RS06270* |  | histidine phosphatase | 4.2 |
| *RHA1_RS06275* |  | hypothetical protein | 4.7 |
| *RHA1_RS06280* | *nadD* | nicotinic acid mononucleotide adenylyltransferase | 3.2 |
| *RHA1_RS09230* |  | triacylglycerol lipase | -16.4 |
| *RHA1_RS11535* |  | lipase | -3.0 |
| *RHA1_RS12225* |  | lipase | 3.1 |
| *RHA1_RS16675* |  | esterase/lipase | -3410 |
| *RHA1_RS16680* |  | cyclohexanone monooxygenase | -6150 |
| *RHA1_RS19560* |  | lipase | -3.6 |
| *RHA1_RS24610* |  | lipase | -2.9 |
| *RHA1_RS26605* |  | lipase | -8.5 |
| *RHA1_RS29850* |  | lipase | 2.8 |
| *RHA1_RS31160* |  | lipase | -10.2 |
| *RHA1_RS34950* |  | lipase | 12.7 |
| *RHA1_RS36250* |  | lipase | 5.3 |
| *RHA1_RS37130* |  | phospholipase | -2.4 |
| *RHA1_RS37595* |  | lipase | 7.5 |
| *RHA1_RS37600* |  | lipase | 8.1 |
| **Central metabolism** | | | |
| Methylmalonyl-CoA pathway | | | |
| *RHA1_RS07065* |  | glyoxalase | 5.4 |
| *RHA1_RS35295* | *mutA* | methylmalonyl-CoA mutase | 8.1 |
| *RHA1_RS35300* | *mutB* | methylmalonyl-CoA mutase | 10.6 |
| *RHA1_RS35305* |  | arginine/ornithine ABC transporter ATPase | 6.3 |
| *RHA1_RS39765* |  | methylmalonyl-CoA carboxyltransferase | 4.1 |
| Pyruvate dehydrogenase | | | |
| *RHA1_RS05795* | *aceE* | pyruvate dehydrogenase E1 | 5.8 |
| *RHA1_RS07665* | *pdhA1* | pyruvate dehydrogenase E1, α subunit | -2.3 |
| *RHA1_RS07670* |  | 2-oxoisovalerate dehydrogenase, β subunit | 1.2 |
| *RHA1_RS07675* |  | branched-chain alpha-keto acid dehydrogenase subunit E2 | 1.3 |
| *RHA1_RS07680* |  | MFS transporter | 1.5 |
| *RHA1_RS10445* | *dldH1* | dihydrolipoyl dehydrogenase | 5.8 |
| *RHA1_RS12550* | *aceE2* | pyruvate dehydrogenase E1 | 8.0 |
| *RHA1_RS16020* |  | pyruvate dehydrogenase E1 | 4.3 |
| *RHA1_RS16375* |  | hypothetical protein | 15.7 |
| *RHA1_RS16380* | *dldH4* | dihydrolipoamide dehydrogenase | 39.5 |
| *RHA1_RS16385* |  | branched-chain α-keto acid dehydrogenase subunit E2 | 14.2 |
| *RHA1_RS16390* | *pdhB2* | 2-oxoisovalerate dehydrogenase, β subunit | 25.2 |
| *RHA1_RS16395* | *pdhA3* | pyruvate dehydrogenase E1, α subunit | 23.9 |
| *RHA1_RS27135* | *dldH5* | FAD-dependent pyridine nucleotide-disulfide oxidoreductase | -2.1 |
| *RHA1_RS31870* | *pycA* | pyruvate carboxylase | -1.8 |
| *RHA1_RS31875* |  | methyltransferase | -1.5 |
| Pentose phosphate and Entner-Doudoroff pathways | | | |
| *RHA1_RS07250* |  | phosphoketolase | -2.7 |
| *RHA1_RS11560* |  | transcriptional regulator | 4.3 |
| *RHA1_RS11565* |  | ketohydroxyglutarate aldolase | 14.7 |
| *RHA1_RS11570* | *edd* | phosphogluconate dehydratase | 9.0 |
| *RHA1_RS11575* | *zwf2* | glucose-6-phosphate 1-dehydrogenase | 1.3 |
| *RHA1_RS27000* | *fba* | fructose-bisphosphate aldolase | 5.3 |
| *RHA1_RS27160* | *pgi* | glucose-6-phosphate isomerase | 4.6 |
| *RHA1_RS27530* | *zwf3* | glucose-6-phosphate 1-dehydrogenase | -2.8 |
| *RHA1_RS28640* | *glpX* | fructose 1,6-bisphosphatase | 2.3 |
| *RHA1_RS35055* | *pgl* | 6-phosphogluconolactonase | 1.4 |
| *RHA1_RS35060* |  | oxidoreductase | 1.8 |
| *RHA1_RS35065* | *zwf4* | glucose-6-phosphate 1-dehydrogenase | 2.7 |
| *RHA1_RS35070* | *tal* | transaldolase | 1.7 |
| *RHA1_RS35075* |  | transketolase | 3.0 |
| **Glyceroneogenesis** | | | |
| *RHA1_RS01950* |  | CoA transferase | 3.5 |
| *RHA1_RS01955* |  | aldehyde dehydrogenase | 5.6 |
| *RHA1_RS01960* |  | transporter | 4.4 |
| *RHA1_RS01965* |  | hypothetical protein | 5.4 |
| *RHA1_RS01970* |  | hypothetical protein | 6.8 |
| *RHA1_RS11710* | *gpsA* | glycerol-3-phosphate dehydrogenase | -5.6 |
| *RHA1_RS12215* |  | betaine-aldehyde dehydrogenase | -13.3 |
| *RHA1_RS14915* |  | aldehyde dehydrogenase | 9.5 |
| *RHA1_RS14920* |  | carnitine dehydratase | 18.6 |
| *RHA1_RS19725* |  | mycolyltransferase | 2.4 |
| *RHA1_RS24240* |  | mycolyltransferase | -5.5 |
| *RHA1_RS29620* |  | alcohol dehydrogenase | -4.8 |
| *RHA1_RS29625* |  | histidine kinase | -3.1 |
| *RHA1_RS29630* |  | LuxR family transcriptional regulator | -2.8 |
| *RHA1_RS29635* |  | tetronasin ABC transporter ATP-binding protein | -6.1 |
| *RHA1_RS31815* | *gpsA* | glycerol-3-phosphate dehydrogenase | 3.4 |
| *RHA1_RS31820* |  | cystathionine gamma-lyase | 3.3 |
| *RHA1_RS31825* | *ddl* | D-alanine--D-alanine ligase | 1.5 |
| *RHA1_RS35045* | *secG* | preprotein translocase subunit SecG | 9.4 |
| *RHA1_RS35050* | *ppc* | phosphoenolpyruvate carboxylase | 5.9 |
| *RHA1_RS43090* | *aldH2* | aldehyde dehydrogenase | -2.2 |
| **Fatty acid biosynthesis and β-oxidation pathways** | | | |
| *RHA1_RS05805* | *fabD* | malonyl CoA-ACP transacylase (FAS II) | 13.3 |
| *RHA1_RS05810* | *acpP* | acyl carrier protein (FAS II) | 10.8 |
| *RHA1_RS05815* | *fabF* | 3-oxoacyl-ACP synthase (FAS II) | 10.2 |
| *RHA1_RS05820* | *pccB* | propionyl-CoA carboxylase, β subunit (FAS II) | 11.0 |
| *RHA1_RS06910* | *acpS* | 4'-phosphopantetheinyl transferase (FAS II) | 48.3 |
| *RHA1_RS06915* | *fas* | 3-oxoacyl-ACP synthase (FAS I) | 115 |
| *RHA1_RS14290* |  | acetyl-CoA carboxylase | 18.2 |
| *RHA1_RS14295* |  | CoA transferase | 38.1 |
| *RHA1_RS14725* |  | CoA ligase | 51.3 |
| *RHA1_RS14730* |  | acyl-CoA dehydrogenase | 85.8 |
| *RHA1_RS14735* | *fabG* | 3-oxoacyl-ACP synthase (FAS II) | 15.6 |
| *RHA1_RS14740* |  | LysR family transcriptional regulator | 2.9 |
| *RHA1_RS20530* |  | acetyl-CoA carboxylase | 133 |
| *RHA1_RS25435* |  | dehydratase | -5.7 |
| *RHA1_RS25440* | *fabG* | 3-ketoacyl-ACP reductase (FAS II) | -5.8 |
| *RHA1_RS28800* |  | acyl-CoA dehydrogenase | -28.9 |
| *RHA1_RS31290* |  | acyl-CoA dehydrogenase | -36.9 |
| *RHA1_RS31295* |  | butyryl-CoA dehydrogenase | -47.6 |
| *RHA1_RS36275* |  | acyl-CoA dehydrogenase | -4.1 |
| *RHA1_RS42100* |  | hypothetical protein | 7.0 |
| *RHA1_RS42105* |  | acyl-CoA synthetase | 6.0 |
| *RHA1_RS42110* |  | oxidoreductase | 15.5 |
| *RHA1_RS42115* |  | hypothetical protein | 7.4 |
| *RHA1_RS42120* |  | hypothetical protein | 4.0 |
| *RHA1_RS42125* |  | phenylacetate--CoA ligase | 6.4 |
| *RHA1_RS42130* |  | aldehyde dehydrogenase | 5.4 |
| *RHA1_RS42135* |  | ferredoxin | 4.5 |
| *RHA1_RS42140* |  | cytochrome P450, C-terminal | 3.8 |
| *RHA1_RS42555* | *accC2* | acetyl-CoA carboxylase, biotin carboxylase subunit | 11.0 |

^a^Shading delineates genes in the same operon. Not all the genes in a given operon had P-values < 0.05.
